# Undrained shear behavior of silty sand with a constant state parameter considering initial stress anisotropy effect

**DOI:** 10.1038/s41598-023-50901-y

**Published:** 2024-01-26

**Authors:** Peipei Li, Chen Zhu, Xiaodong Pan, Bin Lv, Kun Pan

**Affiliations:** College of Civil Engineering, Zhejiang University of Technology, Hangzhou, 310014 China

**Keywords:** Ocean sciences, Engineering

## Abstract

Field observations in sedimentation and erosion-prone areas indicate that most natural sand deposits may contain a certain amount of non-plastic fines and are often under anisotropic stress conditions. A series of triaxial compression tests were performed on clean and silty sand with fines content *f*_c_ ranging from 0 to 20% at an initial mean effective stress of *p*_0_′ = 100 kPa and varying consolidation conditions to understand the impact of initial stress anisotropy on undrained shear behavior. The results indicate that the state parameter *ψ* is a superior predictor for characterizing the responses of sand-fines mixtures compared to the global void ratio and relative density. A comparison of the behavior of clean and silty sand with a constant *ψ* (= − 0.03) confirms that the sample with 10% *f*_c_ exhibits the strongest dilation and greatest shear resistance, irrespective of the consolidation conditions. It is also demonstrated that the initial stress anisotropy with a comparably higher static stress ratio *η*_s_ typically diminishes the shear strength of mixtures. However, the influence of initial stress anisotropy on soil stiffness is not unilateral. The sample consolidated to a negative *η*_s_ is stiffer than that under isotropic consolidation, while the presence of a positive *η*_s_ leads to a decrease in the secant Young*'*s modulus.

## Introduction

The undrained shear behavior of sandy deposits, including pore pressure generation, strength, and deformation characteristics, is critical for geotechnical engineers. This information is essential for designing foundations and retaining structures, evaluating slope stability, and analyzing potential liquefaction during earthquakes. Field observations in sedimentation and erosion-prone areas such as riverbanks, coastal regions, and deltas indicate that most natural and man-made sand deposits, such as hydraulic fills, contain a certain amount of non-plastic silty fines^1–4^. The presence of silty fines in sand significantly impacts the soil’s structural and mechanical behavior, including skeleton, strength, stability, and liquefaction susceptibility. Many laboratory tests have been conducted to examine the undrained shear behavior of sand‒silt mixtures, accounting for multiple factors, such as grain size distribution, type of fines, confining pressure, and loading history^5–8^. However, the results of these tests appear to be contradictory since the effect of non-plastic fines on undrained shear strength could be either beneficial or detrimental, depending on the density state and fines content (*f*_c_).

The void and skeleton structure of sand‒silt mixture are significantly altered with the increasing amount of silty fines added to the host sand. When *f*_c_ is low, the behavior of silty sand is mainly controlled by the sand matrix as the coarse grains form the soil skeleton. However, when *f*_c_ becomes large, the silt matrix composed of fines dominates the soil behavior. This type of transition represents the existence of the threshold fine content (*f*_c,th_), which is related to the fundamental change in the skeleton and void distribution and varies in a relatively narrow range in different tested mixtures, i.e., *f*_c,th_ = 20‒35%^7,9,10^. The value of *f*_c,th_ can be determined by a few experimental approaches through a back-analysis of the limiting void ratios, monotonic and cyclic strength data^11,12^ or using an empirical method based on the following equation^13^

1 $$f_{{\text{c,th}}} = 0.4\left( {\frac{1}{1 + \exp (0.5 - 0.13\chi )} + \frac{1}{\chi }} \right),$$

where *χ* = *D*_10_/*d*_50_ is the particle size ratio, in which *D*_10_ is the diameter of coarse grains at which 10% of sample is finer and* d*_50_ is the mean particle diameter of finer grains. The global void ratio (*e*) and global relative density (*D*_r_) have been widely used to characterize the shear strength of mixtures; however, no consensus has yet been reached. For example, tests on silty sand with a constant *e* indicate a decrease in liquefaction resistance, compared with that of clean sand^10,14,15^. Meanwhile, Sadek and Saleh^16^ and Dash et al.^9^ recommended that in comparison with the adoption of a constant *e* that would induce changes in the soil density, maintaining a constant *D*_r_ is a more legitimate approach for characterizing the mechanical behavior of silty sand. According to Singh^17^, Polito and Martin^15^, and Kim et al.^18^, tests performed at a constant *D*_r_ showed either an unchanged or a moderate increase/decrease in shear strength. Consequently, concerns have been raised about the legitimacy of the conventional *e* and *D*_r_ in describing the response of such mixed soil, and a hypothesis that fines may fill the voids between coarse grains without participating in the force transfer has been put forward. Several studies^19–21^ employed the skeleton void ratio (*e*_s_) to capture this effect, which was developed by Thevanayagam et al.^22^ into the concept of the equivalent skeleton void ratio (*e*_s,eq_) in accounting for the secondary role of fines in force transfer. Bensoula et al.^23^ and Porcino and Diano^24^ further proposed the equivalent relative density in evaluating the liquefaction potential of mixtures contain a wider range of *f*_c_ by replacing *e* with *e*_s,eq_ when calculating *D*_r_. In recent decades, there has been an increased interest in utilizing the state parameter *ψ* to assess the state dependent behavior of silty sand^14,25–27^, which is defined by Been and Jefferies^28^ as the difference between the current *e* and the critical state void ratio *e*_c_ at the same mean effective stress *p'*. A positive *ψ* value at the onset of undrained shearing is generally indicative of contractive behavior, whereas a negative *ψ* value typically signifies dilation and strain hardening. To further define the undrained shear behavior of mixed soils in a coherent manner, Chiu and Fu^29^ and Rahman et al.^8^ proposed a framework that combines the state concept and *e*_s,eq_ by introducing the equivalent granular state parameter *ψ*_eq_. A key step in doing that is to obtain a best-fit parameter that make the critical state data of the clean and silty sand with varying* f*_c_, when plotted in the *e*_s,eq_–*p'* plane, fall within a narrow band that represents a single critical state line (CSL). It is important to note that the CSL for the host sand is no longer unique and is dependent on *f*_c_, thereby violating the principle of the critical state soil mechanics that stipulates the existence of a unique CSL for a given soil.

The preceding experimental studies mainly focused on isotropically consolidated samples. However, silty sand deposits in the field are often under an anisotropic stress condition, sustaining a static deviatoric or shear stress prior to subsequent undrained shearing. Since the studies by Mohamad and Dobry^30^ and Hyodo et al.^31^, numerous studies have been performed to investigate the initial stress anisotropy of sand and its influence on the subsequent shear behavior, such as the elastic deformation characteristics, effective stress path, and shear strength^32–38^. According to Sivathayalan and Ha^32^ and Chu et al.^39^, the initial stress anisotropy may cause the sand become more contractive with more pronounced strain softening. Lashkari et al.^35^ and Fakharian et al.^38^ further examined the effects of initial stress anisotropy on the onset of instability, phase transformation, and critical states, and provided useful information highlighting the anisotropic fabric evolution of the sand during shearing. Nonetheless, few studies have investigated the initial stress anisotropy effect on the undrained shear behavior of silty sand with a varying amount of fines, and a consensus has yet to be reached. Bobei et al.^40^ and Rabbi et al.^41^ examined the undrained shear characteristics of a natural silty sand under both isotropic and anisotropic (*K*_0_) consolidation conditions. They concluded that the static liquefaction potential shows a unique relationship to *ψ*, regardless of the type of consolidation, whereas the shear strength at characteristic states shows slightly different relationships with *ψ* for isotropic and anisotropic consolidation. Using a similar laboratory experimental procedure, Lade and Yamamuro^42^ proposed a simplified approach to analyze the liquefaction potential of silty sand slopes, such as mine tailings and spoil heaps. The effect of initial stress anisotropy on the structural and mechanical properties of sand has also been thoroughly examined by measuring small-to-large strain parameters along various stress paths^43,44^. Based on series of high-quality experimental results, some new conceptions and models were proposed to quantify the shear modulus and damping ratio of samples subjected to anisotropic loading conditions, which has been verified using the results of systematic bender element, resonant column, and hollow cylinder experiments on different types of soil mixtures^37,45–47^.

These preliminary findings emphasize the need for a more comprehensive investigation into the role of initial stress anisotropy in the shear behavior of silty sand at varying *f*_c_. On the other hand, only a few studies have investigated the adequacy of state indices, such as *e*, *D*_r_, and *ψ*, in characterizing the mixtures’ responses, and diverse views exist as to whether the increasing *f*_c_ has a detrimental or beneficial influence. More importantly, it is necessary to further explore how the presence of fines and initial stress anisotropy alters the shear strength and stiffness of sand simultaneously, which is the primary objective of the present study. Thus, a series of undrained triaxial compression tests were conducted on saturated clean and silty sand with *f*_c_ varying from 0 to 20%. The fundamental shear behavior of isotropically consolidated samples was examined by considering different indices, and constant value of *ψ* was used as a comparative benchmark to investigate the effect of initial stress anisotropy. Based on experimental observations of stress–strain behavior and pore pressure development, the strength and stiffness characteristics of silty sand were comprehensively presented and interpreted.

## Materials and methods

**Figure 1:**
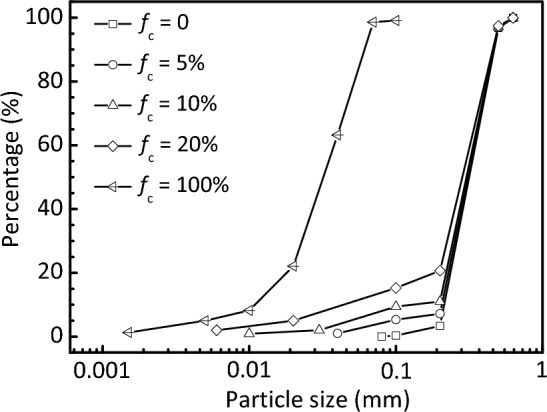
Particle size distribution of the test materials.

The material tested in this study was Fujian sand, a type of Chinese standard sand composed of sub-angular to sub-rounded silica grains. The sand grains were ground into non-plastic fines with particle diameters primarily ranging from 2 to 75 μm. The particle size distribution curves and basic physical properties of the test materials are shown in Fig. 1 and Table 1, respectively. The silty sand sample that is formed by mixing crushed fines with the host sand was controlled at *f*_c_ = 5%, 10%, and 20%. The maximum void ratio (*e*_max_) was determined using Method B in ASTM D4254-16^48^ with a cylindrical tube, and the minimum void ratio (*e*_min_) was measured through vibratory table tests^49^. Although the procedures were recommended for samples with small fines fractions, they were extended in testing mixed soils at a higher *f*_c_ to achieve a consistent comparison^11,15^. The dry proctor test method^7,11^ was also adopted to measure *e*_min_ of the silty sand with* f*_c_ > 10% in the present study. In agreement with the findings of Polito and Martin^15^, the proctor test yields a similar *e*_min_ value to that produced by the vibration method. The results show that the limiting void ratios first decrease and then increase with increasing *f*_c_, and the lowest values appear at around *f*_c_ = 30%, which can be roughly regarded as *f*_c,th_. This value is comparable with that of *f*_c,th_ = 29% determined from the empirical approach using Eq. (1), which has been verified and widely adopted^10,50^.

**Table 1:** Index properties of the test materials.

Fines content *f*_c_ (%)	Mean particle diameter *D*_50_ (mm)	Uniformity coefficient *U*_c_	Specific gravity *G*_s_	Maximum dry density *ρ*_dmax_ (g/cm^3^)	Minimum dry density *ρ*_dmin_ (g/cm^3^)	Maximum void ratio *e*_max_	Minimum void ratio *e*_min_
0	0.350	1.7	2.632	1.660	1.382	0.904	0.586
5	0.343	1.8	2.632	1.728	1.408	0.869	0.523
10	0.336	2.7	2.634	1.798	1.419	0.856	0.465
20	0.315	6.0	2.634	1.921	1.434	0.837	0.371
100	0.034	3.4	2.634	–	–	–	–

Tests were performed utilizing an automated triaxial testing system, as described by Zhou et al.^51^. Samples of approximately 50 mm in diameter and 100 mm in height were prepared via the moist tamping method, which avoids segregation between sand and fine particles^26,52^. The oven-dried sand, mixed with de-aired water to achieve 5% moisture content, was compacted in three layers into a split mold using a small hammer. After removing the split mold, the actual dimensions of the sample and its volumetric strain during preparation and consolidation were measured to calculate the actual density state, which is also determined through the back-analysis procedure by measuring the final water content of the sample at the end of tests^53^. The samples were completely saturated with Skempton’s *B*-values above 0.95 and then isotropically or anisotropically consolidated to a specific static deviatoric stress *q*_s_ (= *σ*_v_ − *σ*_h_, where *σ*_v_ and *σ*_h_ are the vertical and horizontal normal stress, respectively) at an initial mean effective stress of *p*_0_*'* = 100 kPa. The controlled void ratio after consolidation is achieved by a trial procedure. To achieve the same range of void ratio as samples undergoing isotropic consolidation, the sample that will be anisotropically consolidated is intended to be densely or loosely deposited at the preparation stage, depending on its volume change tendency during anisotropic consolidation. If the void ratio of a sample is still out of the desired range, it will be discarded. In fact, the test series selected and shown below are the best outcome in terms of the samples’ density for comparison purpose. Then strain-controlled triaxial compression tests were conducted under undrained conditions by applying the monotonic deviatoric stress at a strain rate of 0.1%/min, which was widely adopted by Hyodo et al.^31^, Murthy et al.^6^, Pan et al.^54^, and Porcino et al.^55^ to evaluate the undrained shear response of both clean and silty sand.

Table 2 summarizes all the monotonic triaxial conditions investigated in this study, as designated by state indices (*e*, *D*_r_, and *ψ*), stress variables (*σ*_v_, *σ*_h_, and *q*_s_), and stress anisotropy factor *η*_s_ (= *q*_s_/*p*_0_*'*), which are classified into four series based on the controlled *f*_c_. The results obtained from the benchmark tests in each series were compared to preliminarily examine the suitability of *e*, *D*_r_, and *ψ* in characterizing the undrained shear behavior of isotropically consolidated samples. Subsequently, tests addressing different *η*_s_ levels were performed on anisotropically consolidated samples under a constant *ψ* (= − 0.03) condition.

**Table 2:** Summary of undrained triaxial tests (*p*_0_*'* = 100 kPa).

Series	*f*_c_ (%)	*e*	*D*_r_ (%)	*ψ*	*σ*_v_ (kPa)	*σ*_h_ (kPa)	*q*_s_ (kPa)	*η*_s_
I	0	0.749	48.7	− 0.125	100	100	0	0
		0.782	38.4	− 0.092	100	100	0	0
		0.812	28.9	− 0.062	100	100	0	0
		0.826	24.5	− 0.048	100	100	0	0
		0.845	18.6	− 0.029	100	100	0	0
		0.844	18.9	− 0.03	100	100	0	0
		0.844	18.9	− 0.03	127	87	40	0.4
		0.844	18.9	− 0.03	140	80	60	0.6
		0.844	18.9	− 0.03	73	113	− 40	− 0.4
II	5	0.702	48.3	− 0.11	100	100	0	0
		0.733	39.3	− 0.079	100	100	0	0
		0.784	24.6	− 0.028	100	100	0	0
		0.804	18.9	− 0.008	100	100	100	0
		0.826	12.4	0.014	100	100	0	0
		0.782	25.1	− 0.03	100	100	0	0
		0.782	25.1	− 0.03	127	87	40	0.4
		0.782	25.1	− 0.03	140	80	60	0.6
		0.782	25.1	− 0.03	73	113	− 40	− 0.4
III	10	0.686	43.5	− 0.065	100	100	0	0
		0.714	36.3	− 0.037	100	100	0	0
		0.734	31.2	− 0.017	100	100	0	0
		0.782	18.9	0.031	100	100	0	0
		0.721	34.5	− 0.03	100	100	0	0
		0.721	34.5	− 0.03	127	87	40	0.4
		0.721	34.5	− 0.03	140	80	60	0.6
		0.721	34.5	− 0.03	73	113	− 40	− 0.4
IV	20	0.608	49.1	− 0.037	100	100	0	0
		0.625	45.5	− 0.02	100	100	0	0
		0.639	42.5	− 0.006	100	100	0	0
		0.656	38.8	0.011	100	100	0	0
		0.615	47.6	− 0.03	100	100	0	0
		0.615	47.6	− 0.03	127	87	40	0.4
		0.615	47.6	− 0.03	140	80	60	0.6
		0.615	47.6	− 0.03	73	113	− 40	− 0.4

## Results

### Undrained shear behavior of isotropically consolidated samples

**Figure 2:**
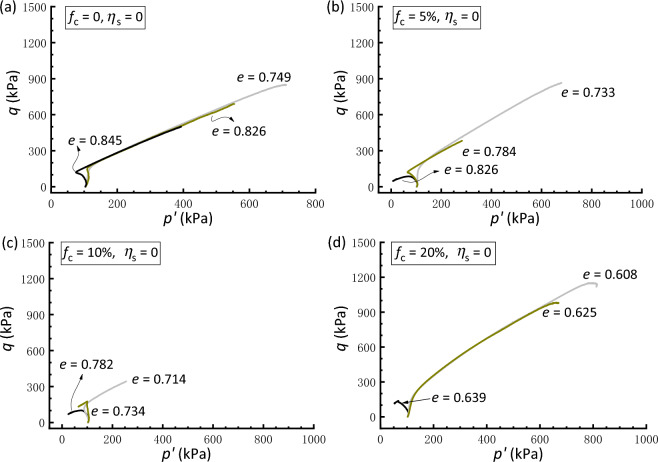
Effective stress paths of isotropically consolidated samples with varying void ratios: (**a**) *f*_c_ = 0; (**b**) *f*_c_ = 5%; (**c**) *f*_c_ = 10%; (**d**) *f*_c_ = 20%.

**Figure 3:**
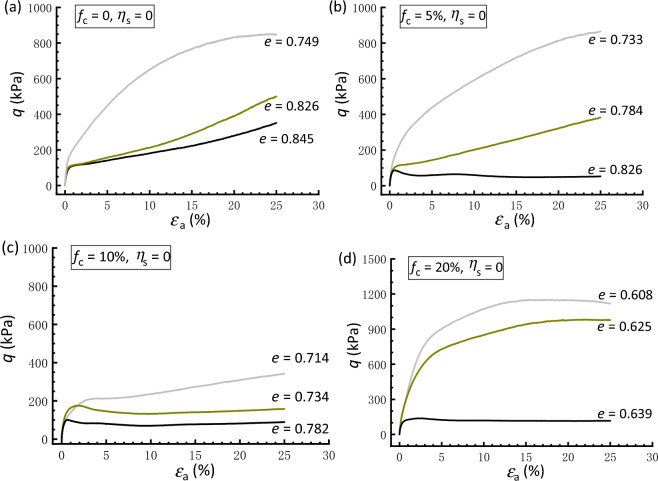
Stress strain curves of isotropically consolidated samples with varying void ratios: (**a**) *f*_c_ = 0; (**b**) *f*_c_ = 5%; (**c**) *f*_c_ = 10%; (**d**) *f*_c_ = 20%.

Figures 2 and 3 illustrate the effective stress path and stress–strain curve, respectively, of isotropically consolidated samples with* f*_c_ = 0, 5%, 10%, and 20%. These data show the effect of the global void ratio *e* after consolidation on the undrained shear behavior. As shown in Fig. 2a, the clean sand with *e* = 0.749 shows predominate dilation, whereas the sample with *e* = 0.845 exhibits an initial contractive phase, followed by dilative behavior. In between is the case of *e* = 0.826, in which the phase-transformation state^56^ that divides the contractive and dilative responses can also be found. In Fig. 3a, all the three clean sand samples display stable strain-hardening behavior toward the critical state. Similarly, for the silty sand a given *f*_c_, a decrease in *e* leads to a shift from a tendency to contract to a tendency to dilate (Fig. 2), accompanied by a transition from strain-softening to strain-hardening behavior (Fig. 3).

**Figure 4:**
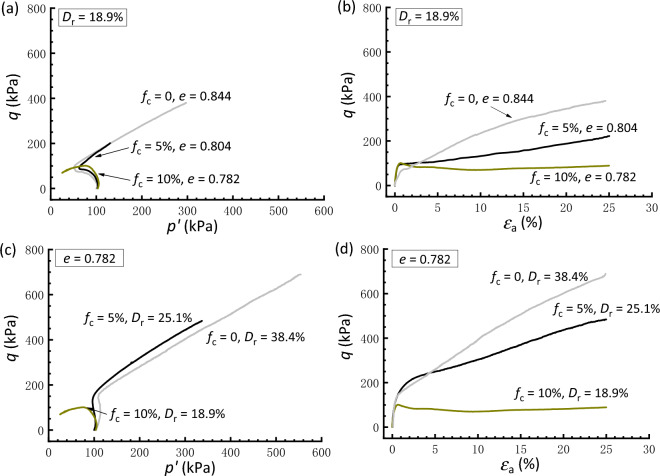
Comparisons of responses of isotropically consolidated samples with a constant relative density or void ratio: (**a,c**) effective stress paths; (**b,d**) stress strain curves.

Figure 4 presents a comparison between the responses of clean and silty sand under isotropic consolidation. The basis for the comparison of samples in Fig. 4a,b is the post-consolidation relative density (*D*_r_ = 18.9%). It is shown that the addition of silty fines results in a decrease in shear strength and an increase in the degree of strain softening compared to the clean sand, although the void ratio declines with increasing *f*_c_. When comparing the responses of samples having a constant *e* (= 0.782), as shown in Fig. 4c,d, the clean sand also exhibits a very dilative response, with a marked and stable increase in strength compared with that of silty sand. In fact, the behavior of silty sand at a lower *f*_c_ is primarily controlled by the sand matrix because the silty fines may fill the voids formed by coarse grains with less participation in the force transfer mechanism.

**Figure 5:**
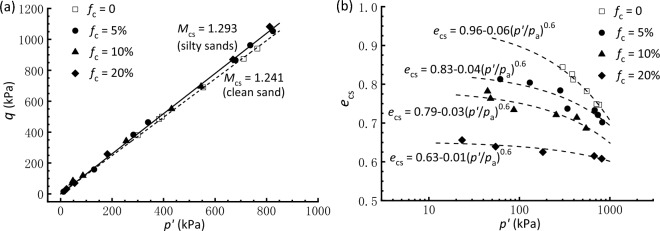
Critical state lines in (**a**) *q*-*p*′ plane and (**b**) *e*-*p*′ plane.

In most of the tests conducted in this study, the axial strain developed over 25% and the rate of variation in deviatoric stress at that strain level was relatively small; such a state is postulated close enough and can be used to approximate the critical state. The critical state data for clean and silty sand are displayed on *q*-*p'* and *e*-log*p'* planes in Fig. 5a,b, respectively. The plot shows that the critical state stress points in *q*-*p'* plane fall within a narrow band that can be represented by straight lines passing through the origin

2 $$q = M_{{{\text{cs}}}} \cdot p^{\prime},$$

where *M*_cs_ is the critical state stress ratio relating to the critical state friction angle *ϕ*_cs_ as

3 $$\sin \phi_{{{\text{cs}}}} = \frac{{3M_{{{\text{cs}}}} }}{{6 + M_{{{\text{cs}}}} }}.$$

A detailed scrutiny of Fig. 5a shows that the clean Fujian sand has a *ϕ*_cs_ value of 30.9°, being comparable with that obtain from Yang and Wei^52^ on the same sand and less than the value for Toyoura sand that is more angular. For the sand mixed with 5–20% fines, Fig. 5a shows a slightly higher *ϕ*_cs_ of 32.1°, although it appears insensitive to the increasing fines contents. This is mainly due to the presence of crushed fines that are angular with irregular geometry and implies that the friction angle of a mixed soil is affected by the shape of both coarse and finer particles. Thus, the shape characteristics of the tested host sand and fines, including the aspect ratio, sphericity, and roundness, should be examined in a quantitative way and further compared with other types of finer and coarse grains in future, which may allow a better understanding of their macro-scale properties that are potentially associated with the particle shape.

Compared with the CSL in the *q*-*p'* plane that is insensitive to *f*_c_ variation, the critical state locus in the *e*-log*p'* plane shown in Fig. 5b descends as *f*_c_ increases up to 20%. This observation aligns with that reported by Thevanayagam et al.^22^, Murthy et al.^6^, and Yang et al.^25^ on several different silty sands. Of note, the critical state locus on the semi-log form is not a straight line but instead a curved line that can be described through a power function of the form proposed by Li and Wang^57^

4 $$e_{{{\text{cs}}}} = e_{\Gamma } - \lambda \left( {\frac{{p^{\prime}}}{{p_{{\text{a}}} }}} \right)^{\xi } ,$$

where *e*_cs_ is the critical state void ratio,* p*_a_ denotes atmospheric pressure (101 kPa), *e*_Γ_, *λ*, and *ξ* are fitting parameters. The least-square regression is employed to estimate the expression for each mixture’s CSL (Fig. 5b), assuming that the power-law exponent *ξ* is constant and equal to 0.6 in line with Yang et al.’s^25^ finding. The parameters *e*_Γ_ and *λ* exhibit a decreasing trend with increasing* f*_c_. Some scatter exists in the critical state data, especially when *f*_c_ goes up, which may be contributed to the change of achieving uniform sample with increasing *f*_c_ and the inherent variability in the material. Ni et al.^2^ and Chiu and Fu^29^ have noted that further increase in *f*_c_ might lead to an upward movement of the CSL. This signifies the presence of the threshold *f*_c,th_ distinguishing the locations of the CSL of silty sand, which is beyond the range of *f*_c_ (0‒20%) considered this study.

**Figure 6:**
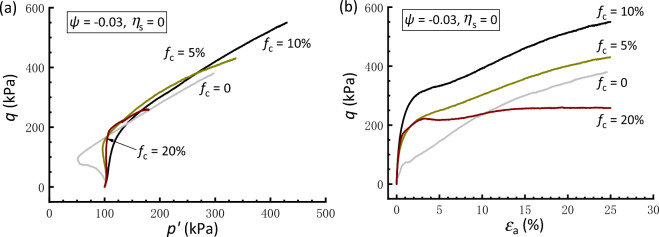
Comparisons of responses of isotropically consolidated samples with a constant state parameter: (**a**) effective stress path; (**b**) stress strain curve.

The behavior of clean and silty sand samples that are packed at a constant *ψ* is shown in Fig. 6, where the value of *ψ* =  − 0.03 is calculated using the CSL specific to *f*_c_ of the sand-fines mixtures, which provides convenience in controlling of samples’ density under the testing conditions. Selecting this value allows the attainment of samples in a loose or medium dense state (*D*_r_ ≈ 20‒50%, as shown in Table 2), neither too loose nor too dense, facilitating subsequent experimental operations and data acquisition. All four isotropically consolidated samples that are undrained sheared from a negative *ψ* exhibit a prominent dilative, stable strain-hardening behavior, which conforms with the framework of critical state soil mechanics. Specifically, the clean sand behaves a contractive phase at the start of undrained shearing, while the degree of contraction decreases as *f*_c_ increases and the sample with* f*_c_ = 10% shows a fully dilative behavior. A transition arises when *f*_c_ increases to 20%, leading to a slightly contractive initial phase for the sample. Correspondingly, the shear strength at the critical state increases first and then decreases with increasing *f*_c_, with the sample at 10%* f*_c_ manifesting the highest shear strength. As granular materials in nature, the sand‒silt mixtures comprising discrete particles exhibit complex structure and mechanical behaviors during the loading process. Shire et al.^58,59^ investigated the micro-structure and micro-properties of granular mixtures under isotropic compression. It has been shown that for a mixture containing a comparatively lower* f*_c_, the finer grains fill voids left by the coarse ones without separating them, such that the latter sustaining the strong force chains will be significantly reinforced by a large number of fines around them. As* f*_c_ further increases to a critical fraction, some of the fines tend to separate the coarse particles from one another, leading to the soil matrix being less resistant to the external forces. This may account for the above phenomenon that samples with 10% and 20% *f*_c_ behave a notable deviation in the stress–strain behavior. Compared with the dramatic differences observed in soil behavior under constant *e* or *D*_r_ (Fig. 4), the overall analogous behavior shown in Fig. 6 implies that *ψ* is a comparatively superior predictor for synthesizing the undrained shear behavior of clean and silty sand.

**Figure 7:**
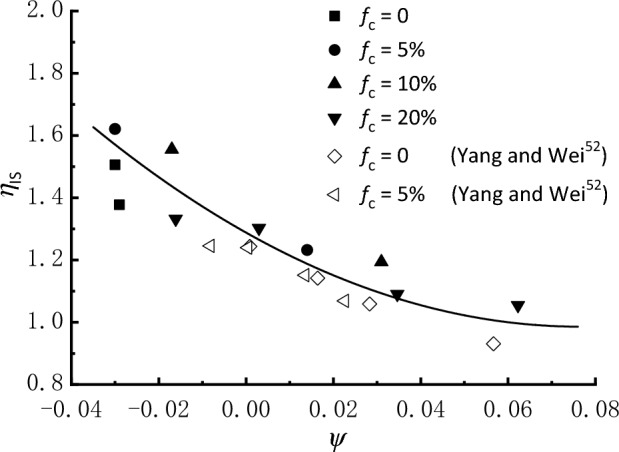
Relation between *η*_IS_ and *ψ* for sand–fines mixtures.

The instability state (IS) representing the peak stress point is a striking feature associated with the strain-softening behavior of sand. In Fig. 7, the stress ratio at the onset of IS, *η*_IS_ = *q*_IS_/*p*_IS_*'*, is plotted against *ψ*; also included are the data from Yang and Wei^52^ on the similar test material for comparison. The variations in data points indicate that the trend of *η*_IS_ with respect to *ψ* is sensitive to the amount of fines. Nevertheless, there exist fairly good correlations between *η*_IS_ and *ψ*, in accordance with which *η*_IS_ decreases with *ψ* reported by Yang and Wei^52^, Lashkari et al.^35^, and Fakharian et al.^38^, implying that the instability is triggered at a lower stress ratio for samples with higher contractive tendency.

### Effect of initial stress anisotropy on strength and stiffness characteristics

**Figure 8:**
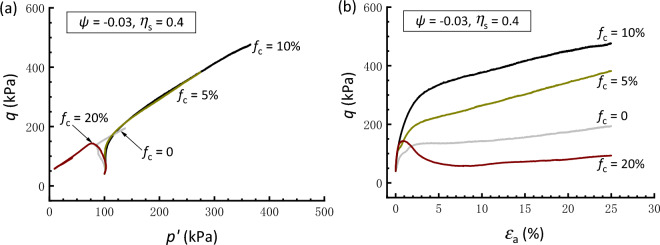
Responses of anisotropically consolidated samples with *η*_s_ = 0.4: (**a**) effective stress path; (**b**) stress strain curve.

**Figure 9:**
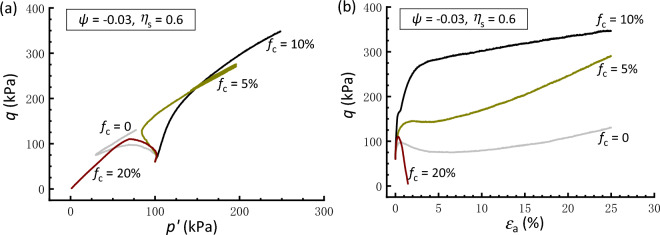
Responses of anisotropically consolidated samples with *η*_s_ = 0.6: (**a**) effective stress path; (**b**) stress strain curve.

**Figure 10:**
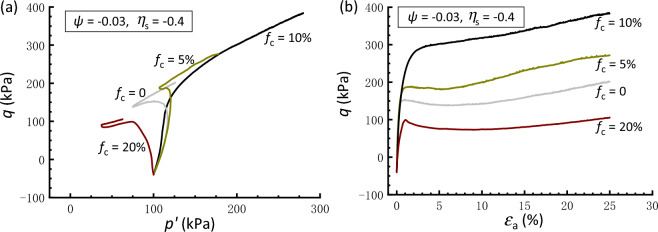
Responses of anisotropically consolidated samples with *η*_s_ = − 0.4: (**a**) effective stress path; (**b**) stress strain curve.

The above results demonstrate the effectiveness of the constant *ψ* approach in the comparative study of the soil mixtures having* f*_c_ values ranging from 0 to 20%. Therefore, the following interpretations regarding the initial stress anisotropy effect are based on tests conducted under the same *ψ* value of − 0.03. Figures 8, 9 and 10 present the effective stress paths and stress–strain curves of clean and silty sand samples that are anisotropically consolidated to different *η*_s_. In Fig. 8, the clean sand subjected to *η*_s_ = 0.4 behaves a contraction-to-dilation manner, while samples mixed with 5% and 10% *f*_c_ show a more dilative behavior. At *f*_c_ = 20%, the sample displays a highly contractive response and achieves a transient minimum shear strength known as the quasi-steady state^60^ prior to dilation and strain hardening toward the critical state. The scenario that the sample with 10%* f*_c_ has the strongest dilation and highest strength is compatible with that observed in isotropically consolidated samples (Fig. 6). Similar observations can be drawn for the sample subjected to *η*_s_ = 0.6 and − 0.4, as depicted in Figs. 9 and 10, respectively. Note that the situation where *η*_s_ =  − 0.4 corresponds to an initial extensional static stress, which is a less investigated but particular point of interest^36^. Moreover, it is found that a comparably higher *η*_s_ level leads to a deterioration in the monotonic shear behavior of the sand-silt mixtures. For example, Fig. 9 shows that the silty sand with *f*_c_ = 20% that undergoes anisotropic consolidation to *η*_s_ = 0.6 is characterized by fully contractive and strain-softening behaviors. This type of behavior, typically associated with the instability, is commonly referred to as static liquefaction^61^.

**Figure 11:**
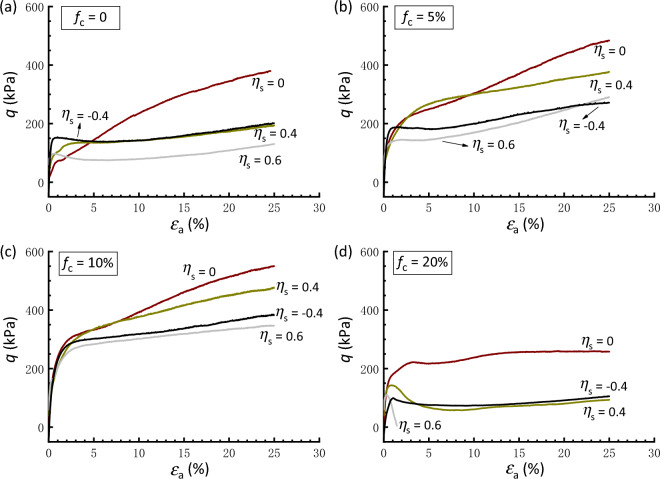
Stress strain curves of samples with varying degrees of initial stress anisotropy at a constant state parameter: (**a**) *f*_c_ = 0; (**b**) *f*_c_ = 5%; (**c**) *f*_c_ = 10%; (**d**) *f*_c_ = 20%.

Figure 11 further presents the stress strain curve of samples with varying degrees of initial stress anisotropy but with a constant state parameter (*ψ* =  − 0.03). Regardless of *f*_c_ levels (0‒20%) considered herein, the sample under isotropic consolidation (*η*_s_ = 0) exhibits a marked stable strain-hardening behavior, in comparison to that undergoes initial stress anisotropy. More specifically, Fig. 11a,d clearly show that samples behave from a strain-hardening type response to a strain-softening type response, as the degree of stress anisotropy increases. The presence of the initial stress anisotropy also has a weakened effect on the undrained shear strength, as illustrated in detail in the following discussion.

**Figure 12:**
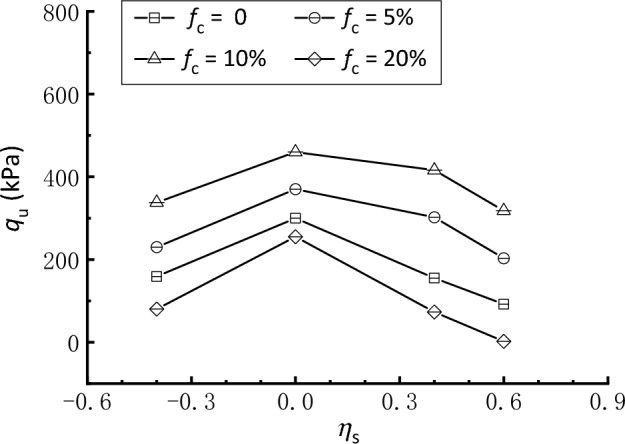
Undrained shear strength of clean and silty sand under different consolidation conditions.

The undrained shear strength represented by *q*_u_ is determined by the peak deviatoric stress for strain-softening type response or the mobilized deviatoric stress at a strain level of 15% for strain-hardening type response. Figure 12 illustrates the variations of *q*_u_ of clean and silty sand with the initial stress anisotropy represented by *η*_s_. For each *f*_c_ considered in this study, *q*_u_ has an increasing and then decreasing trend as *η*_s_ increases from − 0.4 to 0.6 and reaches its maximum value at *η*_s_ = 0, indicating that the initial stress anisotropy has a detrimental effect on the strength of both clean and silty sand. This trend deviates somewhat from Kato et al.^62^, Georgiannou and Konstadinou^63^, and Pan et al.^54^, who reported that anisotropic consolidation with *σ*_v_ > *σ*_h_ at a lower *η*_s_ level leads to an increase in the shear strength of sand. It is also shown that the addition of silty fines can either enhance or reduce the shear strength of sand, depending on* f*_c_. Specifically, the trend of *q*_u_ against *η*_s_ shifts upward as *f*_c_ increases to 10% and then moves downward dramatically with further increases in *f*_c_. Thus, the curves of *f*_c_ = 10% and 20% constitute the upper and lower bounds, respectively. This signifies that there exists a critical* f*_c_ between 10 and 20%, at which although most of the fines are confined within voids, some of them separate the coarse particles from one another, increasing the fragility of the soil^58,64^. This is essentially different from *f*_c,th_ determined above.

The significant differences in the strength characteristics between clean and silty sand under different anisotropic consolidation, as shown in Fig. 12, are intimately related to the particle packing and arrangement of the mixtures. Through the discrete element method (DEM), numerous studies have been performed to investigate the influence of fines and stress anisotropy on the structure and mechanical properties of granular materials (see Minh et al.^65^; Shire et al.^59^; Zhou et al.^66^). It is recognized that grains under gravity may cause their long axes to orient horizontally, making the sample become weaker in triaxial extension than in compression. This may explain the decrease in shear strength for samples with an extensional static deviatoric stress (i.e., a negative *η*_s_). When the sample is anisotropically consolidated with a comparatively higher (compressional) static stress, more grains tend to lie on the horizontal direction^54^, especially for the sample with crushed fines that have more elongated particles. Consequently, the silty sand under anisotropic consolidation features a higher degree of anisotropy than the isotropically consolidated sample owing to the preferential orientations of particles, leading to the former being more contractive and susceptible to shear failure. Nevertheless, a micromechanical study through direct grain-scale observations should be further undertaken to better understand the underlying mechanisms.

**Figure 13:**
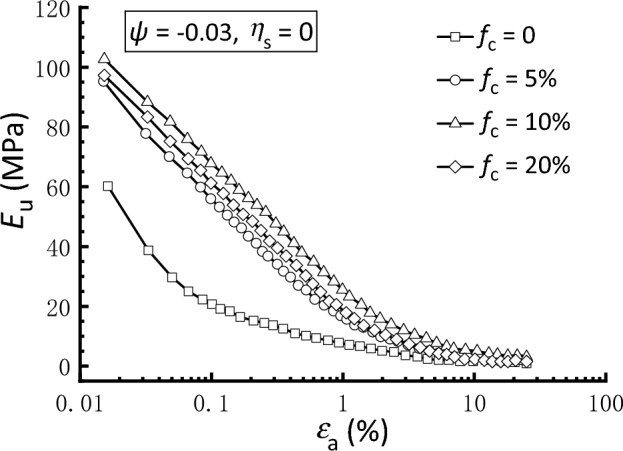
Undrained Young’s modulus degradation curves of samples under isotropic consolidation.

Under undrained triaxial loading, the soil stiffness is usually quantified in terms of the undrained Young’s modulus, *E*_u_, which is defined as the secant slope of the deviatoric stress–strain curve^67^. In Fig. 13, variations in *E*_u_ with the associated axial strain are plotted on a logarithmic scale for isotropically consolidated samples with varying *f*_c_. Overall, a considerable degradation in *E*_u_ is observed for the strain levels considered herein. As shown, the stiffness degradation curve of the clean sand is located on the lower part of the figure, whereas curves of *f*_c_ = 5‒20% are closely arranged on the upper portion. This indicates that the silty sand has significantly higher stiffness than that of clean sand under isotropic consolidation; however, this strengthening effect is insensitive to the amount of fines. It can also be observed in Fig. 13 that the difference in stiffness data between the clean and silty sand gradually narrows as strain increases, and the stiffness degradation curves tend to coincide when the axial strain surpasses 1%.

**Figure 14:**
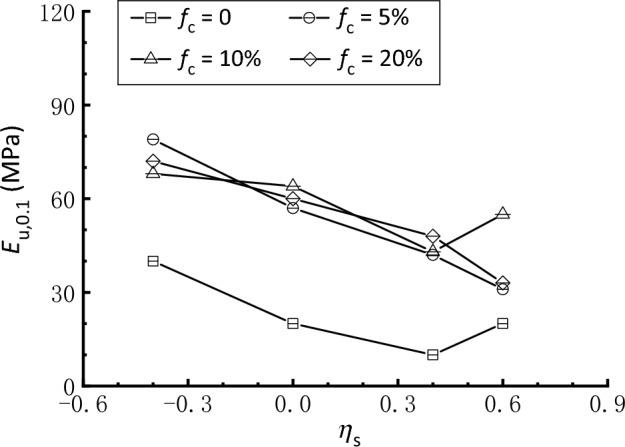
Undrained Young’s modulus at *ε*_a_ = 0.1% (*E*_u,0.1_) of clean and silty sand under different consolidation conditions.

According to Clayton^67^ and Pan et al.^54^, the secant stiffness at *ε*_a_ = 0.1% is significant for analyzing soil‒structure interaction since strain levels associated with geotechnical structures, including spread foundations, retaining walls, and tunnels, always fall within this range. Figure 14 shows a summary of the undrained Young’s modulus *E*_u_ at *ε*_a_ = 0.1% (*E*_u,0.1_) for clean and silty sand under different consolidation conditions. Similar to Fig. 13, Fig. 14 reveals again that the addition of 5‒20% silty fines to clean sand has a beneficial influence on the stiffness, irrespective of *η*_s_ levels. However, the *E*_u,0.1_ values for silty sand samples under specific consolidation condition do not display a systematic shift as* f*_c_ transitions from 5 to 20%, indicating that the effect of *f*_c_ on stiffness is non-monotonic. It is also shown that both the clean and silty sand have an overall decreasing trend of *E*_u,0.1_ as *η*_s_ increases from − 0.4 to 0.6 with two exceptions, which are samples with* f*_c_ = 0 and 10% subjected to *η*_s_ = 0.6. As a result, it is observed that anisotropic consolidation with *σ*_v_ < *σ*_h_ (*η*_s_ =  − 0.4) enhances the soil stiffness during triaxial compression in comparison to isotropic consolidation (*η*_s_ = 0), whereas anisotropic consolidation with *σ*_v_ > *σ*_h_ (*η*_s_ = 0.4 and 0.6) has an adverse effect. These findings are consistent with those reported by Yamashita et al.^68^ and Pan et al.^54^, who discovered that static deviatoric stress on one side (compression or extension) might reduce the stiffness when the sample is subsequently sheared on the same side but strengthen it when the sample is sheared on the other side.

## Conclusion

A series of undrained triaxial compression tests were conducted on clean and silty sand with the fines content *f*_c_ ranging from 0 to 20% under either isotropic or anisotropic consolidation to an initial mean effective stress of* p*_0_*'* = 100 kPa. The adequacy of state indices, such as the global void ratio *e*, relative density *D*_r_, and state parameter *ψ* in characterizing the behavior of isotropically consolidated samples was examined. The results of tests considering the effect of initial stress anisotropy under a constant *ψ* (= − 0.03) condition were presented and analyzed, with a focus on the strength and stiffness characteristics. The following main conclusions can be drawn from this study:

1. Comparing the undrained shear behavior of clean and silty sand using different state indices reveals varying perspectives on the effect of silty fines. While substantial differences in soil behavior under constant *e* or *D*_r_ are evident, the similar overall behavior with a constant *ψ* implies that *ψ* is a comparatively superior predictor for synthesizing the responses of sand-fines mixtures, which also provides fairly good correlations to the instability stress ratio.
2. The state of axial strain developed to 25% is used to approximate the critical state of mixtures. A unique critical state line is obtained in the *q*-*p'* plane for samples mixed with 5‒20% fines, yielding a critical state friction angle of 32.1°, which is slightly larger than that determined from the clean sand. This is mainly due to the presence of crushed fines that are more angular with irregular geometry than the host sand. Conversely, the critical state locus in the *e*-log*p'* plane is non-unique and can be described with a power function, exhibiting a declining tendency as *f*_c_ increases up to 20%.
3. Under a constant *ψ* condition, the silty sand with 10%* f*_c_ has the strongest dilation and highest shear resistance. A comparably higher static stress ratio *η*_s_ typically leads to a deterioration in the monotonic shear behavior. Specifically, the undrained shear strength increases and then decreases as *η*_s_ increases from − 0.4 to 0.6 and peaks at *η*_s_ = 0, indicating that the initial stress anisotropy has a detrimental effect on the strength of both clean and silty sand. A grain-scale interpretation is made to provide a better understanding of these macro-observations; however, further laboratory experiments covering a wide range of *f*_c_ and *ψ* and micromechanical studies through discrete element simulations are favorably undertaken to examine the underlying mechanisms.
4. The initial stress anisotropy may act positively or negatively on the undrained shear stiffness quantified by the secant Young’s modulus, depending on the direction of *η*_s_. The stiffness of mixtures in triaxial compression may reduce if the sample undergoes a compressional *η*_s_ but increase if it acts on the extension side. Moreover, the addition of 5‒20% silty fines to clean sand has a favorable impact on the stiffness, regardless of the *η*_s_ levels considered herein.

## Data Availability

Some or all data, models, and code that support the findings of this study are available from the corresponding author upon reasonable request.
